# Irinotecan in patients with relapsed or cisplatin-refractory germ cell cancer: a phase II study of the German Testicular Cancer Study Group

**DOI:** 10.1038/sj.bjc.6601176

**Published:** 2003-09-09

**Authors:** T Powles, J Shamash, D Berney, R T D Oliver

**Affiliations:** 1 Department of Medical Oncology, St Bartholomew's Hospital, 1st Floor, King George V Building, West Smithfield, London ECIA 7BE, UK

**Sir**,

We read with interest the phase II results presented by Kollmannsberger *et al* (2002b), which recorded no responses to single-agent irinotecan delivered in a dose of 300–350 mg/m^−2^ every 3 weeks. This led the authors to conclude that further evaluation of irinotecan in patients with germ cell cancer could not be recommended. We note that other recent single-agent phase II studies in germ cell tumours (oxaliplatin, paclitaxel and gemcitabine (Sandler *et al*, 1998; Bokemeyer *et al*, 1999; Kollmannsberger *et al*, 2002a)) revealed very low objective response rates (Table 1

**Table 1:** Chemotherapy for relapsed/refractory germ cell tumours

**Reference**	***n***	**Treatment**	**Prior exposure to platinum (cycles)**	**Prior high-dose CT (%)**	**Refractory/absolute refractory disease (%)**	**Response rate PR/CR (%)**	**Overall survival (months)**
Kollmannsberger *et al*	32	Oxaliplatin	7	78	85	13	5
Sandler *et al*	18	Paclitaxel	>6	16	NA	11	3.5
Bokemeyer *et al*	31	Gemcitabine	7	71	54	19	6
Hinton *et al*	28	Gemcitabine	>6	36	36	21	8
		Paclitaxel					
Bockemeyer *et al*	15	Irinotecan	6	86	74	0	3
IPO	9	Irinotecan	7	22	22	55	>11
		Paclitaxel					
		Oxaliplatin					

CT=chemothotherapy; PR=partail remission; CR=complete remission.

) The patients treated in the studies are invariably heterogenous; however, most have proved refractory to at least two lines of cisplatin-based therapy and many have received prior high-dose therapy. Since the number of durable responses following high-dose therapy is of the region of only 4% and that 86% of patients in Kollmannsberger's manuscript had received high-dose therapy, we feel that the results are not particularly surprising. The median survival in all these studies is remarkably similar and in the region of 3–6 months. However, when gemcitabine and paclitaxel were given in combination, the overall survival was improved (Hinton *et al*, 2002).

We have recently reported in this journal that there is an increased level of topoisomerase-1 expression in viable tumour samples from residual masses postchemotherapy (Berney *et al*, 2002). Moreover, *in vitro* studies suggest that the active metabolite of irinotecan (SN38) may be active in germ cell tumours. Evidence for this comes from both published data (Ueno *et al*, 2002) and currently unpublished work in progress, from our institution. SN38 appears to have impressive efficacy in both sensitive and platinum-resistant testicular cancer cell lines (sensitive testicular lines: IC_50_=1.8–7.4 nm, resistant testicular line: IC_50_=7.0 nm). It is for this reason that we have felt that the investigation of combination chemotherapy based on irinotecan was warranted. We have been evaluating a combination chemotherapy regimen using oxaliplatin 100 mg/ m^−2^ on 1 one, irinotecan 200 mg/ m^−2^ on day 1 and paclitaxel 80 mg/ m^−2^ on days 1, 8 and 15 (IPO), the treatment being repeated every 21 days. To date we have treated nine patients. Two had absolutely refractory disease to cisplatin and two had relapsed following high-dose chemotherapy. Four patients have had complete remissions and two have marker negative partial remissions. Currently, the median survival for this cohort has not been reached, with a median follow-up of 11 months (range 4–14). Both patients with absolute refractory disease are currently progression free 11 and 12 months after treatment.

We conclude that combining drugs with relatively low single-agent activity, leads to improved results in these patients. This effect has also been shown when oxaliplatin and irinotecan were combined in fluorouracil-resistant colorectal carcinoma, as compared to their single-agent activity (Scheithauer *et al*, 1999). Our results are certainly comparable with those of Nomoto *et al*, who also used irinotecan in combination with cisplatin in relapsed disease; however, details regarding platinum sensitivity of their patients were not given (Nomoto *et al*, 2002). These *in vitro* and *in vivo* observations lead us to question whether single-agent phase II studies, conducted in a highly refractory patient population may best be able to identify potentially useful cytotoxics, and certainly a negative result must be accepted with some degree of caution.
